# Increased risk of suicide among patients with social anxiety disorder

**DOI:** 10.1017/S204579602500006X

**Published:** 2025-02-25

**Authors:** Han-Ting Wei, Shih-Jen Tsai, Chih-Ming Cheng, Wen-Han Chang, Ya-Mei Bai, Tung-Ping Su, Tzeng-Ji Chen, Mu-Hong Chen

**Affiliations:** 1 Department of Psychiatry, Taipei Veterans General Hospital, Taipei, Taiwan; 2 Department of Psychiatry, College of Medicine, National Yang Ming Chiao Tung University, Taipei, Taiwan; 3 Department of Psychiatry, Taipei City Hospital, Branch of Linsen, Chinese Medicine, and Kunming, Taipei, Taiwan; 4 Department of Psychiatry, General Cheng Hsin Hospital, Taipei, Taiwan; 5 Department of Family Medicine, Taipei Veterans General Hospital, Taipei, Taiwan; 6 Institute of Hospital and Health Care Administration, National Yang Ming Chiao Tung University, Taipei, Taiwan; 7 Department of Family Medicine, Taipei Veterans General Hospital, Hsinchu Branch, Hsinchu, Taiwan

**Keywords:** psychiatric comorbidities, social anxiety disorder, suicide, Taiwan

## Abstract

**Aims:**

Increasing evidence has established a strong association between social anxiety disorder and suicidal behaviours, including suicidal ideation and suicide attempts. However, the association between social anxiety disorder and suicide mortality remains unclear.

**Methods:**

This study analysed data from 15,776 patients with social anxiety disorder, extracted from a nationwide Taiwanese cohort between 2003 and 2017. Two unexposed groups without social anxiety disorder, matched by birth year and sex in 1:4 and 1:10 ratios, respectively, were used for comparison. Suicide deaths during the same period were examined. Psychiatric comorbidities commonly associated with social anxiety disorder, including schizophrenia, bipolar disorder, major depression, alcohol use disorder (AUD), substance use disorder (SUD), obsessive-compulsive disorder, autism, and attention deficit hyperactivity disorder, were identified.

**Results:**

Time-dependent Cox regression models, adjusted for demographic factors and psychiatric comorbidities, revealed that individuals with social anxiety disorder had an increased risk of suicide (hazard ratio: 3.49 in the 1:4 matched analysis and 2.84 in the 1:10 matched analysis) compared with those without the disorder. Comorbidities such as schizophrenia, bipolar disorder, major depression, AUD, and SUD further increased the risk of suicide in patients with social anxiety disorder.

**Conclusion:**

Social anxiety disorder is an independent risk factor for suicide death. Additional psychiatric comorbidities, including schizophrenia, major affective disorders, and AUD, further increased social anxiety disorder-related suicide risk. Therefore, mental health officers and clinicians should develop targeted suicide prevention strategies for individuals with social anxiety disorder.

## Introduction

Social anxiety disorder, a prevalent yet often overlooked mental disorder, is characterized by an intense fear of embarrassment, rejection, or humiliation in social or public settings, driven by the anticipation of negative evaluation by others (Liebowitz *et al.*, 1985; Nagata *et al.*, 2015; Salari *et al.*, 2024). Social anxiety disorder is a chronic condition that usually manifests in childhood or early adolescence and has a negative effect on social interactions, academic performance, and professional functioning in both children and adults (Liebowitz *et al.*, 1985; Nagata *et al.*, 2015; Salari *et al.*, 2024). Global field studies have estimated that social anxiety disorder affects 4.7% of children, 8.3% of adolescents, and 17% of young adults (Salari *et al.*, 2024). Despite its prevalence, only a small proportion of individuals with social anxiety disorder seek mental consultation or treatment, often delaying seeking assistance until they develop comorbid psychiatric conditions, such as major affective disorders (Liebowitz *et al.*, 1985; Nagata *et al.*, 2015).

Growing evidence has established a strong association between social anxiety disorder and suicidal symptoms, including suicidal ideation and suicide attempts (Buckner *et al.*, 2017; Leigh *et al.*, 2023; Thibodeau *et al.*, 2013). The National Comorbidity Survey Replication and the National Epidemiologic Survey on Alcohol and Related Conditions revealed that individuals with social anxiety disorder had significantly higher odds of experiencing lifetime suicidal ideation (odds ratio [OR], 95% confidence interval [CI]: 2.09, 1.68–2.60; 1.72, 1.58–1.87) and suicide attempts (OR, 2.70, 95% CI: 1.93–3.79; 1.62, 1.42–1.85), irrespective of comorbid major affective disorders, alcohol use disorder (AUD), and substance use disorder (SUD) (Thibodeau *et al.*, 2013). Buckner et al. further identified an indirect link between social anxiety disorder and suicidal ideation, mediated by feelings of thwarted belongingness and perceived burdensomeness, in line with the Interpersonal-Psychological Theory of Suicide (Buckner *et al.*, 2017). A meta-analysis of 16 cross-sectional and prospective studies similarly demonstrated that social anxiety disorder was associated with lifetime suicide attempts (r = 0.10, 95% CI: 0.04–0.15) and current suicidal ideation (r = 0.22, 95% CI: 0.02–0.41) (Leigh *et al.*, 2023). Despite these findings, research has yet to explore the relationship between social anxiety disorder and suicide mortality.

This study obtained data from the Taiwan National Health Insurance Research Database (NHIRD), which comprises data for the entire population of Taiwan (n = 29,077,426), and compared the risk of suicide among patients with social anxiety disorder to that of individuals without social anxiety disorder. Furthermore, the effects of social anxiety disorder-related psychiatric comorbidities, such as bipolar disorder, major depressive disorder, and autism, on suicide risk were evaluated. The primary hypothesis was that social anxiety disorder serves as an independent risk factor for suicide mortality, irrespective of the presence of these psychiatric comorbidities. Furthermore, we hypothesized that patients with social anxiety disorder who also presented with additional psychiatric comorbidities exhibited the highest suicide risk.

## Methods

### Data source

The Health Data Science Center of the Taiwan Ministry of Health and Welfare audits and makes available for research purposes the NHIRD, which includes comprehensive healthcare data on almost 99.7% of Taiwan’s population. To protect individual privacy, individual medical records are kept anonymous in the NHIRD. We linked the Longitudinal Health Insurance Database of the NHIRD, which contains all medical records from 2003 to 2017 of the entire Taiwanese population, and the Database of All-cause Mortality, which includes all-cause mortality records from 2003 to 2017 of the entire Taiwanese population, to analyse the suicide risk among people with social anxiety disorder. The International Classification of Diseases, Ninth or Tenth Revision, Clinical Modification is used in Taiwanese clinical practice (ICD-9-CM [2003–2014] or ICD-10-CM [2015–2017]). Since deidentified data were utilized in this study and no individuals were actively enrolled, the Taipei Veterans General Hospital’s institutional review board authorized the study methodology and waived the need for informed consent. The NHIRD has been used in numerous epidemiological studies in Taiwan (Chen *et al.*, 2013; Cheng *et al.*, 2018; Hsu *et al.*, 2023; Zhang *et al.*, 2021).

### Inclusion criteria for individuals with and without social anxiety disorder

**Figure 1.**
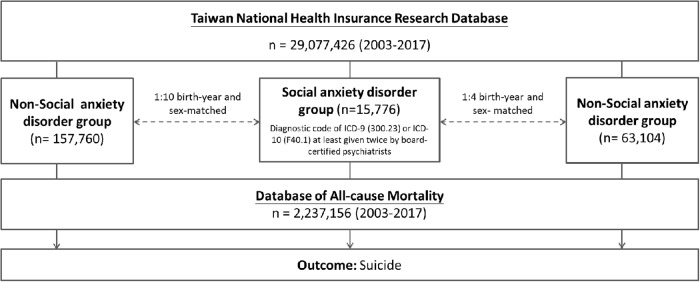
Study flowchart.

This study was a matched retrospective cohort study, and individuals who were diagnosed with social anxiety disorder (the exposed group) were matched to those without social anxiety disorder (the unexposed group). Specifically, the individuals in the social anxiety disorder group were those who had at least two diagnoses of social anxiety disorder (ICD-9-CM code: 300.23 or ICD-10-CM code: F41.1) from board-certified psychiatrists (Fig. 1). A 1:4 exposed and unexposed groups-matched analysis based on birth year and sex was carried out to reduce the confounding effects of age and sex. The unexposed group was selected at random from the entire Taiwanese population after all people who had ever been diagnosed with social anxiety disorder were removed from the database (Fig. 1). In Taiwan, levels 1–4, which represent the most to least urbanized residential areas, were evaluated as a proxy for the availability of healthcare (Liu *et al.*, 2006). The income and urbanicity levels were defined using the most recent data of all individuals. The social anxiety disorder diagnosis was regarded as a time-dependent variable. The time zero was defined as 1 January 2003, for those who were born before 2003 and was defined as the birthdates for those who were born ≥2003, respectively. Suicide was identified between 2003 and 2017 from the Database of All-cause Mortality. For patients with social anxiety disorder and matched individuals without social anxiety disorder, the Charlson Comorbidity Index (CCI) values were calculated during the study period. Every enrolled subject’s systemic health state was determined by evaluating the 22 physical conditions that make up the CCI (Charlson *et al.*, 1987). Furthermore, we further assessed social anxiety disorder-related psychiatric comorbidities with suicide risk, including schizophrenia (ICD-9-CM code: 295 or ICD-10-CM code: F20, F25), bipolar disorder (ICD-9-CM codes: 296 except 296.2, 296.3, 296.9, and 296.82 or ICD-10-CM codes: F30, F31), major depressive disorder (ICD-9-CM codes: 296.2, 296.3, 300.3, 311 or ICD-10-CM codes: F32, F33, F34), OCD (ICD-9-CM code: 300.3 or ICD-10-CM code: F42), attention-deficit hyperactivity disorder (ADHD) (ICD-9-CM code: 314 or ICD-10-CM code: F90), autism (ICD-9-CM codes: 299.0, 299.8, 299.9 or ICD-10-CM codes: F84.0, F84.5, F84.8, F84.9), AUD (ICD-9-CM codes: 291, 303.0, 303.9, 305.0 or ICD-10-CM code: F10), and SUD (ICD-9-CM codes: 292, 304, 305 except 305.0 and 305.1 or ICD-10-CM codes: F11, F12, F13, F14, F15, F16, F18, F19) during the study period, because those comorbid psychiatric disorders are also associated with suicide risk. The study period was defined from 1 January 2003, or birthdates, to 31 December 2017, or death. These psychiatric disorders were diagnosed at least twice by board-certified psychiatrists. Finally, given that increasing the number of people in the comparison improved the statistical power, following the same procedure mentioned above to select the unexposed group, we additionally performed a sensitivity analysis using a 1:10 exposed and unexposed groups-matched analysis to reconfirm our findings (Fig. 1).

### Statistical analysis

For between-group comparisons in grouping data, we employed conditional logistic regressions for nominal variables and repeated measure analyses of variance with general linear models for continuous variables. The hazard ratio (HR) and 95% CI of subsequent suicide between groups were determined using time-dependent Cox regression models with adjustments for sex, birth year, income, level of urbanization, psychiatric comorbidities, and CCI. We investigated the effects of psychiatric comorbidities, such as schizophrenia, bipolar disorder, major depressive disorder, autism, ADHD, OCD, AUD, and SUD, on the suicide risk among people with social anxiety disorder in comparison to the unexposed group using Cox regression models with adjustments for sex, birth year, income, levels of urbanization, and CCI. The proportional hazards assumptions were verified using the log-minus-log plots, resulting in no considerable violation. Finally, given that social anxiety disorder is a female-predominant mental condition (Liebowitz *et al.*, 1985; Nagata *et al.*, 2015; Salari *et al.*, 2024), sex-stratified analyses were performed in the present study. A two-tailed *p* value of <0.05 was considered statistically significant. All data processing and statistical analyses were performed using the Statistical Analysis Software Version 9.1 (SAS Institute, Cary, NC, USA).

## Results

The current study identified 15,776 patients with social anxiety disorder, matching their birth year and sex to 63,104 (1:4 unexposed cohort) and 157,760 (1:10 unexposed cohort) individuals, respectively (Table 1). Of the patients with social anxiety disorder, 1534 (9.72%) had co-occurring schizophrenia, 1490 (9.44%) had bipolar disorder, 9112 (57.76%) had major depressive disorder, 1588 (10.07%) had OCD, 651 (4.13%) had autism, 1571 (9.96%) had ADHD, 316 (2.00%) had AUD, and 381 (2.42%) had SUD (Table 1). Rates of all co-occurring mental disorder diagnoses were significantly higher in the social anxiety disorder group than in the two unexposed groups (Table 1). Patients with social anxiety disorder had higher CCI scores, resided in the more urban area, and had higher income than the two unexposed groups (Table 1).

**Table 1.** Demographic characteristics of patients with social anxiety disorder and matched individuals

	Patients with social anxiety disorder (n = 15,776)	1:4 matched comparison group (n = 63,104)	1:10 matched comparison group (n = 157,760)
Birth Year (n, %)			
−1950	212 (1.3)	848 (1.3)	2120 (1.3)
1951–1960	613 (3.9)	2452 (3.9)	6130 (3.9)
1961–1970	1315 (8.3)	5260 (8.3)	13,150 (8.3)
1971–1980	2586 (16.4)	10,344 (16.4)	25,860 (16.4)
1981–1990	5514 (35.0)	22,056 (35.0)	55,140 (35.0)
1991–2000	4675 (29.6)	18,700 (29.6)	46,750 (29.6)
2000-	861 (5.5)	3444 (5.5)	8610 (5.5)
Male (n, %)	9177 (58.2)	36,708 (58.2)	91,770 (58.2)
Monthly Income (n, %)			
0–1000 USD	4572 (29.0)	19,935 (31.6)	49,849 (31.6)
1001–1800 USD	6716 (42.5)	27,339 (43.3)	68,530 (43.4)
≥1801 USD	4488 (28.5)	15,830 (25.1)	39,381 (25.0)
Level of urbanization (n, %)			
1 (Urban)	3893 (24.7)	12,022 (19.1)	29,855 (18.9)
2	4421 (28.0)	16,340 (25.8)	40,505 (25.7)
3	2751 (17.4)	11,769 (18.7)	29,660 (18.8)
4	3140 (19.9)	14,140 (22.4)	35,654 (22.6)
5 (Rural)	1571 (10.0)	8833 (14.0)	22,086 (14.0)
CCI (n, %)			
0	5574 (35.3)	30,959 (49.1)	77,197 (48.9)
1–2	7619 (48.3)	26,130 (41.4)	65,304 (41.4)
>2	2583 (16.4)	6,015 (9.5)	15,259 (9.7)
Psychiatric comorbidities (n, %)			
Schizophrenia	1534 (9.7)	463 (0.7)	1126 (0.7)
Bipolar disorders	1490 (9.4)	414 (0.7)	1028 (0.7)
Major depressive disorder	9112 (57.8)	2519 (4.0)	6050 (3.8)
OCD	1588 (10.1)	200 (0.3)	474 (0.3)
Autism	651 (4.1)	138 (0.2)	327 (0.2)
ADHD	1571 (10.0)	769 (1.2)	1910 (1.2)
AUD	316 (2.0)	264 (0.4)	661 (0.4)
SUD	381 (2.4)	294 (0.5)	827 (0.5)

OCD: obsessive compulsive disorder; USD: United State dollar; CCI: Charlson Comorbidity Index; ADHD: attention deficit hyperactivity disorder; AUD: alcohol use disorder; SUD: substance use disorder.

Adjusting for sex, birth year, income, level of urbanization, psychiatric comorbidities, and CCI, patients with social anxiety disorder were more likely (HR, 95% CI) to die by suicide than the two unexposed groups, specifically 3.49 (2.02–6.03) in the 1:4 exposed and unexposed groups-matched analysis and 2.84 (1.94–4.17) in the 1:10 exposed and unexposed groups-matched analysis (Table 2).

**Table 2.** Suicide risk between patients with social anxiety disorder and matched individuals

	All sample			Male sample			Female sample		
	Event (n, %)	Suicide rate (per 100,000 person-year)	HR^#^	Event (n, %)	Suicide rate (per 100,000 person-year)	HR^#^	Event (n, %)	Suicide rate (per 100,000 person-year)	HR^#^
			(95% CI)			(95% CI)			(95% CI)
	Analysis 1: 1:4 matched cohort analysis								
Patients with social anxiety disorder	123 (0.78)	52.65	**3.49 (2.02–6.03)**	77 (0.84)	56.64	**3.54 (1.72–7.25)**	46 (0.70)	47.09	**3.14 (1.12–8.81)**
Comparison group	59 (0.09)	6.30	1 (ref)	46 (0.13)	8.44	1 (ref)	13 (0.05)	3.32	1 (ref)
	Analysis 2: 1:10 matched cohort analysis								
Patients with social anxiety disorder	123 (0.78)	52.65	**2.84 (1.94–4.17)**	77 (0.84)	56.64	**2.55 (1.59–4.09)**	46 (0.70)	47.09	**3.63 (1.74–7.56)**
Comparison group	159 (0.10)	6.79	1 (ref)	127 (0.14)	9.33	1 (ref)	32 (0.05)	3.27	1 (ref)

HR: hazard ratio; CI: confidence interval; CCI: Charlson Comorbidity Index.

# adjusting for sex, birth year, income, level of urbanization, psychiatric comorbidities and CCI.

**Bold type** indicates the statistical significance.

Lastly, we evaluated the additional psychiatric comorbidities associated with social anxiety disorder in relation to the suicide risk (Table 3). Both the 1:4 exposed and unexposed groups-matched and 1:10 exposed and unexposed groups-matched analyses revealed a higher risk of suicide death among patients with social anxiety disorder who had schizophrenia (5.96, 3.43–10.34; 4.47, 2.84–7.04), bipolar disorder (5.88, 3.35–10.33; 4.08, 2.59–6.43), major depressive disorder (8.64, 6.05–12.35; 6.67, 4.98–8.93), AUD (7.02, 3.45–14.29; 4.47, 2.40–8.32), or SUD (4.43, 2.13–9.20; 2.91, 1.53–5.53) compared with those without corresponding psychiatric comorbidities (Table 3). Furthermore, only females with social anxiety disorder who were comorbid with OCD (8.49, 2.95–24.43; 5.07, 2.08–12.33) and autism (8.97, 1.71–47.20; 5.51, 1.17–26.07) had a higher suicide risk compared with those without OCD and autism (Table 3).

**Table 3.** Suicide risk between patients with social anxiety disorder with different psychiatric comorbidities and matched individuals

	Analysis 1: 1:4 matched cohort analysis			Analysis 2: 1:10 matched cohort analysis		
Suicide (HR, 95%)^#^	All sample	Male sample	Female sample	All sample	Male sample	Female sample
Comparison group	1.00 (ref.)	1.00 (ref.)	1.00 (ref.)	1.00 (ref.)	1.00 (ref.)	1.00 (ref.)
Patients with social anxiety disorder						
Without schizophrenia	**2.76 (1.76–4.32)**	**2.15 (1.25–3.72)**	**4.83 (2.11–11.06)**	**2.21 (1.57–3.12)**	**1.87 (1.23–2.84)**	**3.26 (1.73–6.14)**
With schizophrenia	**5.96 (3.43–10.34)**	**4.79 (2.48–9.24)**	**10.23 (3.66–28.60)**	**4.47 (2.84–7.04)**	**3.97 (2.31–6.82)**	**6.25 (2.66–14.68)**
Comparison group	1.00 (ref.)	1.00 (ref.)	1.00 (ref.)	1.00 (ref.)	1.00 (ref.)	1.00 (ref.)
Patients with social anxiety disorder						
Without bipolar disorder	**2.68 (1.71–4.20)**	**2.08 (1.20–3.59)**	**4.72 (2.06–10.82)**	**2.12 (1.50–2.99)**	**1.77 (1.17–2.69)**	**3.19 (1.69–6.01)**
With bipolar disorder	**5.88 (3.35–10.33)**	**5.58 (2.83–10.99)**	**6.74 (2.37–19.15)**	**4.08 (2.59–6.43)**	**4.15 (2.41–7.16)**	**3.83 (1.65–8.89)**
Comparison group	1.00 (ref.)	1.00 (ref.)	1.00 (ref.)	1.00 (ref.)	1.00 (ref.)	1.00 (ref.)
Patients with social anxiety disorder						
Without major depressive disorder	**2.67 (1.53–4.67)**	1.72 (0.81–3.68)	**6.06 (2.48–14.85)**	**2.34 (1.39–3.95)**	1.48 (0.72–3.05)	**5.78 (2.62–12.75)**
With major depressive disorder	**8.64 (6.05–12.35)**	**7.34 (4.80–11.23)**	**13.13 (6.61–26.08)**	**6.67 (4.98–8.93)**	**5.65 (3.98–8.01)**	**10.47 (6.04–18.16)**
Comparison group	1.00 (ref.)	1.00 (ref.)	1.00 (ref.)	1.00 (ref.)	1.00 (ref.)	1.00 (ref.)
Patients with social anxiety disorder						
Without OCD	**2.91 (1.88–4.50)**	**2.44 (1.46–4.09)**	**4.53 (1.98–10.40)**	**2.38 (1.71–3.30)**	**2.17 (1.47–3.21)**	**3.01 (1.60–5.66)**
With OCD	**2.33 (1.21–4.50)**	1.13 (0.46–2.78)	**8.49 (2.95–24.43)**	1.78 (1.00–3.19)	1.00 (0.44–2.28)	**5.07 (2.08–12.33)**
Comparison group	1.00 (ref.)	1.00 (ref.)	1.00 (ref.)	1.00 (ref.)	1.00 (ref.)	1.00 (ref.)
Patients with social anxiety disorder						
Without autism	**2.86 (1.85–4.41)**	**2.30 (1.36–3.87)**	**4.79 (2.11–10.88)**	**2.30 (1.66–3.19)**	**2.01 (1.36–2.97)**	**3.19 (1.72–5.93)**
With autism	2.06 (0.61–7.00)	0.78 (0.10–5.94)	**8.97 (1.71–47.20)**	1.73 (0.53–5.63)	0.75 (0.10–5.53)	**5.51 (1.17–26.07)**
Comparison group	1.00 (ref.)	1.00 (ref.)	1.00 (ref.)	1.00 (ref.)	1.00 (ref.)	1.00 (ref.)
Patients with social anxiety disorder						
Without ADHD	**2.90 (1.88–4.48)**	**2.35 (1.40–3.95)**	**4.81 (2.12–10.92)**	**2.33 (1.68–3.23)**	**2.05 (1.38–3.02)**	**3.20 (1.72–5.95)**
With ADHD	1.22 (0.42–3.55)	0.72 (0.16–3.16)	3.50 (0.70–17.47)	1.05 (0.37–2.94)	0.71 (0.17–2.96)	2.56 (0.56–11.63)
Comparison group	1.00 (ref.)	1.00 (ref.)	1.00 (ref.)	1.00 (ref.)	1.00 (ref.)	1.00 (ref.)
Patients with social anxiety disorder						
Without AUD	**2.70 (1.73–4.20)**	**2.16 (1.27–3.69)**	**4.61 (2.01–10.61)**	**2.13 (1.53–2.98)**	**1.88 (1.26–2.80)**	**2.94 (1.56–5.55)**
With AUD	**7.02 (3.45–14.29)**	**4.49 (1.90–10.58)**	**21.38 (5.80–78.84)**	**4.47 (2.40–8.32)**	**3.25 (1.52–6.91)**	**11.88 (3.79–37.22)**
Comparison group	1.00 (ref.)	1.00 (ref.)	1.00 (ref.)	1.00 (ref.)	1.00 (ref.)	1.00 (ref.)
Patients with social anxiety disorder						
Without SUD	**2.78 (1.79–4.31)**	**2.20 (1.29–3.74)**	**4.76 (2.09–10.83)**	**2.20 (1.58–3.07)**	**1.88 (1.26–2.81)**	**3.16 (1.70–5.89)**
With SUD	**4.43 (2.13–9.20)**	**3.68 (1.59–8.51)**	**6.04 (1.29–28.26)**	**2.91 (1.53–5.53)**	**2.73 (1.31–5.68)**	3.49 (0.88–13.78)

OCD: obsessive-compulsive disorder; ADHD: attention deficit hyperactivity disorder; AUD: alcohol use disorder; SUD: substance use disorder.

# Separate Cox regression models with adjustment of sex, birth year, income, level of urbanization, and CCI.

**Bold type** indicates the statistical significance.

## Discussion

The study findings supported the hypothesis that patients with social anxiety disorder had a higher likelihood of dying by suicide than did those without social anxiety disorder, independent of comorbid psychiatric disorders, such as schizophrenia, major affective disorders, AUD, and SUD. Additionally, comorbid schizophrenia, bipolar disorder, major depressive disorder, AUD, and SUD further increased the risk of suicide among patients with social anxiety disorder.

Substantial evidence has demonstrated an association between social anxiety disorder and suicidal ideation and suicide attempts, independent of comorbid major psychiatric disorders (Buckner *et al.*, 2017; Leigh *et al.*, 2023; Thibodeau *et al.*, 2013). This suggests that core symptoms of social anxiety disorder play a crucial role in suicidality (Chiu *et al.*, 2024; Gallagher *et al.*, 2014). A prospective study was conducted on 2,397 adolescents aged 14–24 years to assess symptoms of social anxiety disorder, depression, and suicidal ideation at baseline and follow-up. The results revealed that baseline social anxiety disorder symptoms were associated with suicidal ideation and depressive symptoms 2 years later (Chiu *et al.*, 2024). Buckner et al. indicated a pivotal role of perceived burdensomeness in the association between social anxiety and suicidal ideation, particularly among individuals experiencing increased thwarted belongingness (Buckner *et al.*, 2017). This may indicate that a vicious cycle of social frustration and subsequent social dyscognition fosters the development of suicidal symptoms. In a clinical study, 144 adolescents were evaluated for social anxiety disorder, depression, suicidal ideation, and loneliness during psychiatric hospitalization, with follow-up assessments at 9 and 18 months (Gallagher *et al.*, 2014). Gallagher et al. identified a direct link between social anxiety symptoms at baseline and suicidal ideation at the 18-month follow-up after baseline depressive symptoms and ideation were controlled for (Gallagher *et al.*, 2014). Additionally, they demonstrated that loneliness at the 9-month follow-up mediated this relationship (Gallagher *et al.*, 2014). Rapp et al. noted a direct association between social anxiety disorder and active suicidal ideation and suicide attempts, particularly among ethnic minorities (Rapp *et al.*, 2017). A combined analysis of two community-based family studies, namely the National Institute of Mental Health study and the Cohort Study of Lausanne, revealed that social anxiety disorder in probands was associated with suicide attempts in first-degree relatives (OR: 2.4; 95% CI: 1.7–3.5), even after adjustment for comorbid affective disorders and SUD (Ballard *et al.*, 2019). Ballard et al. reported a familial coaggregation of social anxiety disorder and suicide attempts, indicating a genetic overlap between two conditions (Ballard *et al.*, 2019). Our study is the first to identify social anxiety disorder as an independent risk factor for suicide, regardless of the major psychiatric comorbidities.

Despite the passage of time, social anxiety disorder remains underrecognized in both clinical and nonclinical settings (Liebowitz *et al.*, 1985; Nagata *et al.*, 2015), which may partly account for the relatively small number of individuals identified with social anxiety disorder in this study (n = 15,776). Patients with social anxiety disorder often delay seeking mental health support and treatment until they develop comorbid psychiatric disorders (Liebowitz *et al.*, 1985; Nagata *et al.*, 2015; Salari *et al.*, 2024). This could explain why, in the present study, more than three-quarters of individuals with social anxiety disorder also had other major psychiatric disorders, such as schizophrenia, bipolar disorder, major depressive disorder, AUD, and SUD. Given this high rate of comorbidity, we infer that conditions such as schizophrenia, bipolar disorder, major depressive disorder, AUD, and SUD further elevated the risk of suicide among individuals with social anxiety disorder in the present study (Buckner *et al.*, 2017; Leigh *et al.*, 2023; Thibodeau *et al.*, 2013).

Interestingly, we observed that female but not males with social anxiety disorder and OCD or autism exhibited an elevated suicide risk. Research has demonstrated that females with autism often exhibit more severe autistic symptoms, particularly in social interaction and cognition, than do males with autism (Lai *et al.*, 2023). Another of our studies similarly indicated that females with autism (HR: 4.30, 95% CI: 2.54–7.25) had a higher risk of unnatural-cause mortality, including suicide, than did males with autism (2.06, 1.62–2.63) (Tsai *et al.*, 2023). Additionally, studies reported a positive correlation between symptoms of social anxiety disorder and autism (Gaziel-Guttman *et al.*, 2023; Montaser *et al.*, 2023), suggesting that females with autism experience more severe social anxiety disorder symptoms than do males with autism. The combined effects of autism and social anxiety may be more pronounced in female than in males (Gaziel-Guttman *et al.*, 2023; Montaser *et al.*, 2023; Tsai *et al.*, 2023), which may explain why only females with autism with social anxiety disorder exhibited an elevated risk of suicide in the present study. Furthermore, Asher et al. and Mathes et al. reported that females with social anxiety disorder or OCD exhibited more severe clinical presentations and experienced greater subjective distress compared with their male counterparts (Asher and Aderka, 2018; Mathes *et al.*, 2019). Asher et al. also revealed that males with social anxiety disorder were more likely to seek treatment than were females (Asher and Aderka, 2018). These findings echo our finding of an increased risk of suicide among female but not males with social anxiety disorder and OCD. However, although there were significant differences in HRs between males and females in the subgroups of patients with social anxiety disorder with or without autism or OCD, the 95% CIs still overlapped, which may limit the conclusion of the gender differences in such subgroups. Further studies would be required to elucidate the role of genders in associations between additional psychiatric comorbidities (i.e., autism, OCD) with social anxiety disorder and suicide.

Several limitations of this study should be acknowledged. First, the prevalence of social anxiety disorder may have been underestimated. As noted, social anxiety disorder continues to be widely overlooked in both clinical and nonclinical settings. Additionally, the NHIRD only includes individuals who sought medical consultation and treatment, potentially reflecting a population with more severe clinical presentations. Further research is necessary to determine whether our findings can be generalized to individuals with milder symptoms of social anxiety disorder. Second, in the present study, social anxiety disorder was diagnosed by board-certified psychiatrists at least twice, which increased the diagnostic validity of social anxiety disorder. However, the NHIRD did not have an exact algorithm used to identify cases of social anxiety disorder, which may lead to the possibility of the misclassification. Further cohort studies using the face-to-face diagnostic interview would be necessary to validate our findings. Third, the NHIRD lacks data on psychosocial and environmental factors, personal lifestyle choices, and childhood experiences. The absence of these variables limits our ability to assess their potential effect on the relationship between social anxiety disorder and suicide risk.

In conclusion, patients with social anxiety disorder were at a higher risk of suicide than were those without, regardless of the presence of major psychiatric comorbidities, including schizophrenia, bipolar disorder, major depressive disorder, AUD, and SUD. These comorbidities further increased the risk of suicide among individuals with social anxiety disorder. Notably, autism and OCD increased suicide risk only among females with social anxiety disorder. Mental health professionals and clinicians should prioritize suicide prevention strategies tailored to individuals with social anxiety disorder, particularly those with comorbid major psychiatric disorders.

## Data Availability

The Department of Health and the Bureau of the NHI Program provided and audited the NHIRD with the intention of using it for scientific study (https://www.apre.mohw.gov.tw). Through a formal application that is governed by the Health Data Science Center of the Taiwan Ministry of Health and Welfare, one can access the NHIRD.
